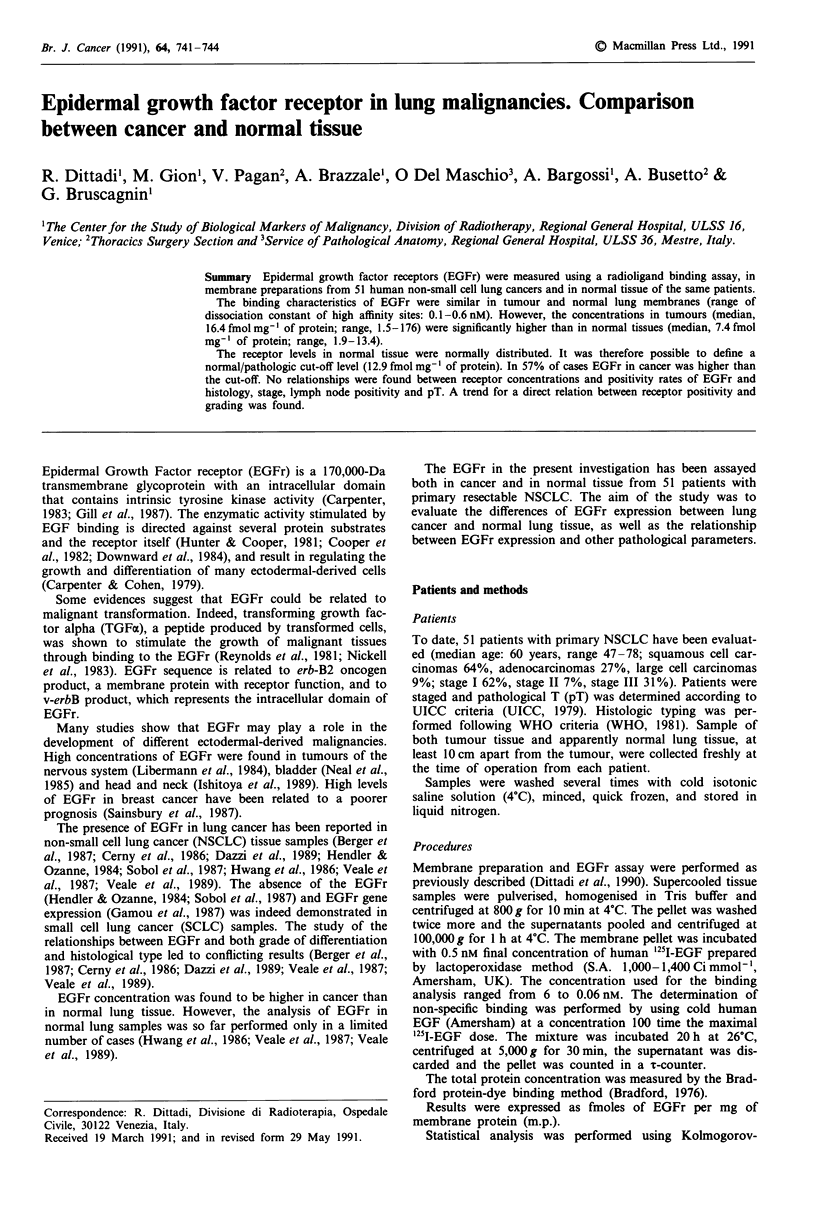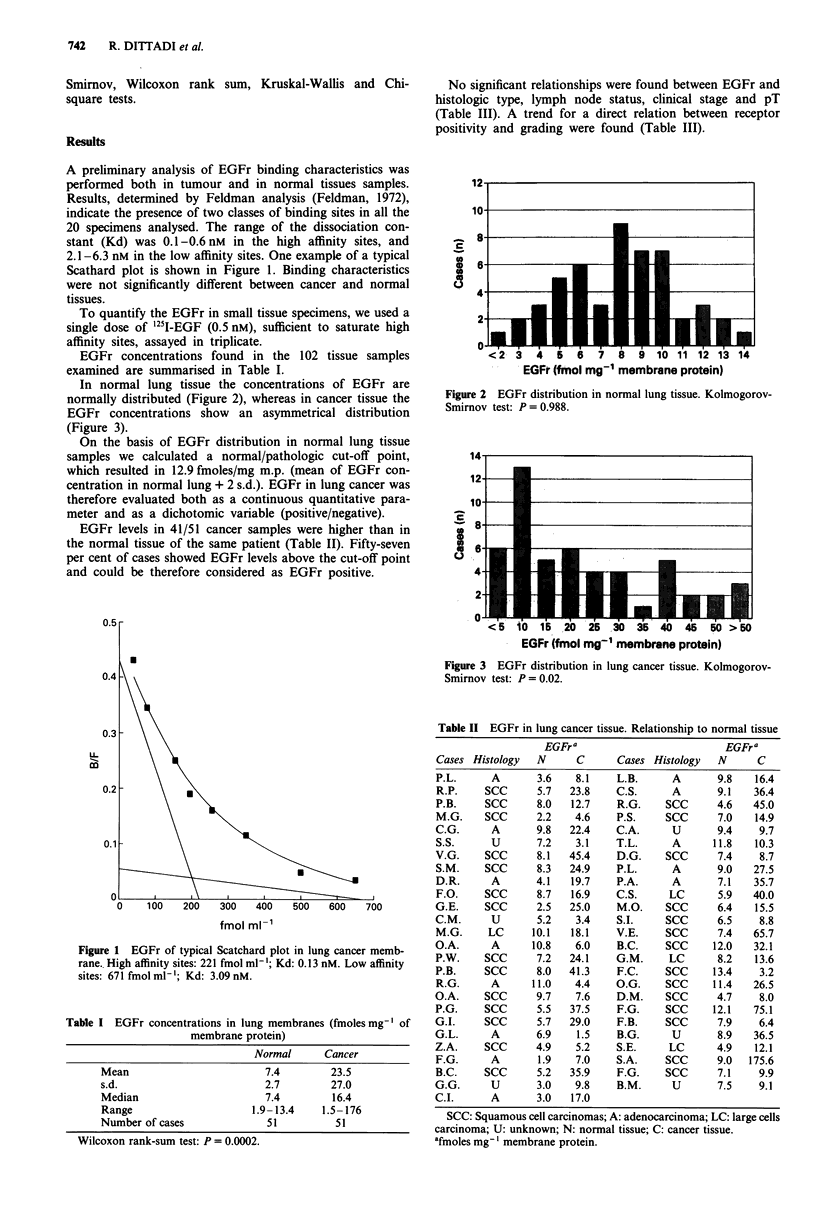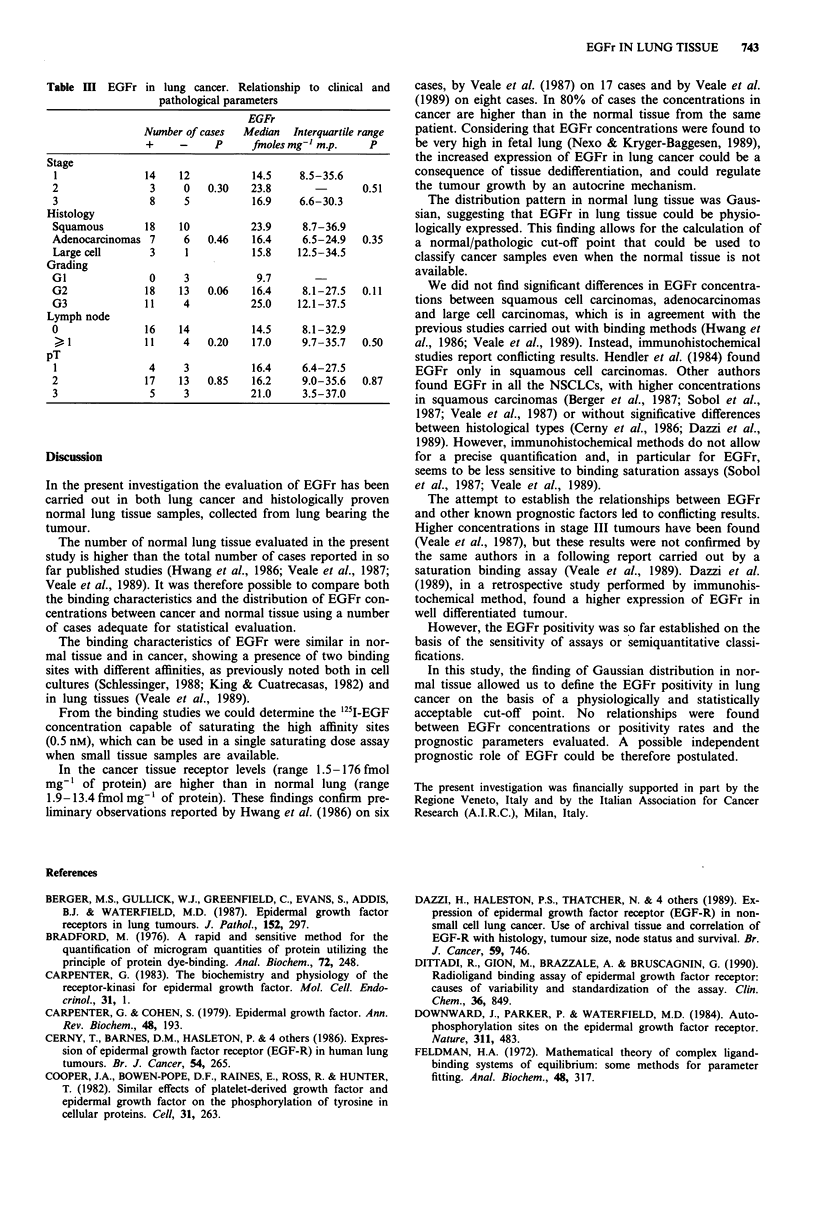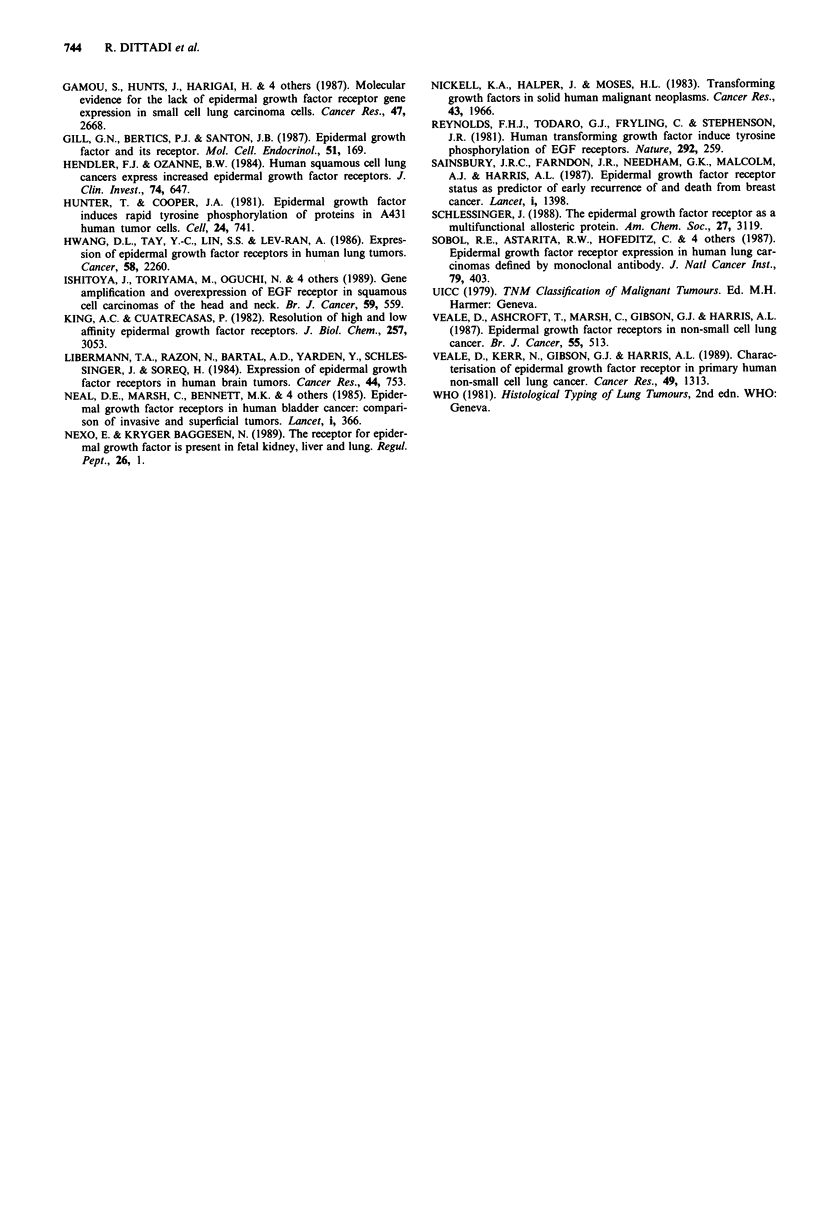# Epidermal growth factor receptor in lung malignancies. Comparison between cancer and normal tissue.

**DOI:** 10.1038/bjc.1991.390

**Published:** 1991-10

**Authors:** R. Dittadi, M. Gion, V. Pagan, A. Brazzale, O. Del Maschio, A. Bargossi, A. Busetto, G. Bruscagnin

**Affiliations:** Cancer for the Study of Biological Markers of Malignancy, Regional General Hospital, Venice, Italy.

## Abstract

Epidermal growth factor receptors (EGFr) were measured using a radioligand binding assay, in membrane preparations from 51 human non-small cell lung cancers and in normal tissue of the same patients. The binding characteristics of EGFr were similar in tumour and normal lung membranes (range of dissociation constant of high affinity sites: 0.1-0.6 nM). However, the concentrations in tumours (median, 16.4 fmol mg-1 of protein; range, 1.5-176) were significantly higher than in normal tissues (median, 7.4 fmol mg-1 of protein; range, 1.9-13.4). The receptor levels in normal tissue were normally distributed. It was therefore possible to define a normal/pathologic cut-off level (12.9 fmol mg-1 of protein). In 57% of cases EGFr in cancer was higher than the cut-off. No relationships were found between receptor concentrations and positivity rates of EGFr and histology, stage, lymph node positivity and pT. A trend for a direct relation between receptor positivity and grading was found.


Br. J. Cancer (1991), 64, 741-744 © Macmillan Press Ltd., 1991

### Epidermal growth factor receptor in lung malignancies. Comparison between cancer and normal tissue

R. Dittadi[1], M. Gion[1], V. Pagan[2], A. Brazzale[1], O Del Maschio[3], A. Bargossi[1], A. Busetto[2] & G. Bruscagnin[1]

[1]*The Center for the Study of Biological Markers of Malignancy, Division of Radiotherapy, Regional General Hospital, ULSS 16, Venice;* [2]*Thoracics Surgery Section and* [3]*Service of Pathological Anatomy, Regional General Hospital, ULSS 36, Mestre, Italy.*

**Summary** Epidermal growth factor receptors (EGFr) were measured using a radioligand binding assay, in membrane preparations from 51 human non-small cell lung cancers and in normal tissue of the same patients. The binding characteristics of EGFr were similar in tumour and normal lung membranes (range of dissociation constant of high affinity sites: 0.1-0.6 nM). However, the concentrations in tumours (median, 16.4 fmol $mg^{-1}$ of protein; range, 1.5-176) were significantly higher than in normal tissues (median, 7.4 fmol $mg^{-1}$ of protein; range, 1.9-13.4).

The receptor levels in normal tissue were normally distributed. It was therefore possible to define a normal/pathologic cut-off level (12.9 fmol $mg^{-1}$ of protein). In 57% of cases EGFr in cancer was higher than the cut-off. No relationships were found between receptor concentrations and positivity rates of EGFr and histology, stage, lymph node positivity and pT. A trend for a direct relation between receptor positivity and grading was found.

Epidermal Growth Factor receptor (EGFr) is a 170,000-Da transmembrane glycoprotein with an intracellular domain that contains intrinsic tyrosine kinase activity (Carpenter, 1983; Gill *et al.*, 1987). The enzymatic activity stimulated by EGF binding is directed against several protein substrates and the receptor itself (Hunter & Cooper, 1981; Cooper *et al.*, 1982; Downward *et al.*, 1984), and result in regulating the growth and differentiation of many ectodermal-derived cells (Carpenter & Cohen, 1979).

Some evidences suggest that EGFr could be related to malignant transformation. Indeed, transforming growth factor alpha (TGFα), a peptide produced by transformed cells, was shown to stimulate the growth of malignant tissues through binding to the EGFr (Reynolds *et al.*, 1981; Nickell *et al.*, 1983). EGFr sequence is related to *erb*-B2 oncogen product, a membrane protein with receptor function, and to v-*erb*B product, which represents the intracellular domain of EGFr.

Many studies show that EGFr may play a role in the development of different ectodermal-derived malignancies. High concentrations of EGFr were found in tumours of the nervous system (Libermann *et al.*, 1984), bladder (Neal *et al.*, 1985) and head and neck (Ishitoya *et al.*, 1989). High levels of EGFr in breast cancer have been related to a poorer prognosis (Sainsbury *et al.*, 1987).

The presence of EGFr in lung cancer has been reported in non-small cell lung cancer (NSCLC) tissue samples (Berger *et al.*, 1987; Cerny *et al.*, 1986; Dazzi *et al.*, 1989; Hendler & Ozanne, 1984; Sobol *et al.*, 1987; Hwang *et al.*, 1986; Veale *et al.*, 1987; Veale *et al.*, 1989). The absence of the EGFr (Hendler & Ozanne, 1984; Sobol *et al.*, 1987) and EGFr gene expression (Gamou *et al.*, 1987) was indeed demonstrated in small cell lung cancer (SCLC) samples. The study of the relationships between EGFr and both grade of differentiation and histological type led to conflicting results (Berger *et al.*, 1987; Cerny *et al.*, 1986; Dazzi *et al.*, 1989; Veale *et al.*, 1987; Veale *et al.*, 1989).

EGFr concentration was found to be higher in cancer than in normal lung tissue. However, the analysis of EGFr in normal lung samples was so far performed only in a limited number of cases (Hwang *et al.*, 1986; Veale *et al.*, 1987; Veale *et al.*, 1989).

Correspondence: R. Dittadi, Divisione di Radioterapia, Ospedale Civile, 30122 Venezia, Italy.
Received 19 March 1991; and in revised form 29 May 1991.

The EGFr in the present investigation has been assayed both in cancer and in normal tissue from 51 patients with primary resectable NSCLC. The aim of the study was to evaluate the differences of EGFr expression between lung cancer and normal lung tissue, as well as the relationship between EGFr expression and other pathological parameters.

### Patients and methods

### *Patients*

To date, 51 patients with primary NSCLC have been evaluated (median age: 60 years, range 47-78; squamous cell carcinomas 64%, adenocarcinomas 27%, large cell carcinomas 9%; stage I 62%, stage II 7%, stage III 31%). Patients were staged and pathological T (pT) was determined according to UICC criteria (UICC, 1979). Histologic typing was performed following WHO criteria (WHO, 1981). Sample of both tumour tissue and apparently normal lung tissue, at least 10 cm apart from the tumour, were collected freshly at the time of operation from each patient.

Samples were washed several times with cold isotonic saline solution (4°C), minced, quick frozen, and stored in liquid nitrogen.

### *Procedures*

Membrane preparation and EGFr assay were performed as previously described (Dittadi *et al.*, 1990). Supercooled tissue samples were pulverised, homogenised in Tris buffer and centrifuged at 800 *g* for 10 min at 4°C. The pellet was washed twice more and the supernatants pooled and centrifuged at 100,000 *g* for 1 h at 4°C. The membrane pellet was incubated with 0.5 nM final concentration of human $^{125}$I-EGF prepared by lactoperoxidase method (S.A. 1,000-1,400 Ci $mmol^{-1}$, Amersham, UK). The concentration used for the binding analysis ranged from 6 to 0.06 nM. The determination of non-specific binding was performed by using cold human EGF (Amersham) at a concentration 100 time the maximal $^{125}$I-EGF dose. The mixture was incubated 20 h at 26°C, centrifuged at 5,000 *g* for 30 min, the supernatant was discarded and the pellet was counted in a τ-counter.

The total protein concentration was measured by the Bradford protein-dye binding method (Bradford, 1976).

Results were expressed as fmoles of EGFr per mg of membrane protein (m.p.).

Statistical analysis was performed using Kolmogorov-

Smirnov, Wilcoxon rank sum, Kruskal-Wallis and Chi-square tests.

### Results

A preliminary analysis of EGFr binding characteristics was performed both in tumour and in normal tissues samples. Results, determined by Feldman analysis (Feldman, 1972), indicate the presence of two classes of binding sites in all the 20 specimens analysed. The range of the dissociation constant (Kd) was 0.1–0.6 nM in the high affinity sites, and 2.1–6.3 nM in the low affinity sites. One example of a typical Scathard plot is shown in Figure 1. Binding characteristics were not significantly different between cancer and normal tissues.

To quantify the EGFr in small tissue specimens, we used a single dose of $^{125}$I-EGF (0.5 nM), sufficient to saturate high affinity sites, assayed in triplicate.

EGFr concentrations found in the 102 tissue samples examined are summarised in Table I.

In normal lung tissue the concentrations of EGFr are normally distributed (Figure 2), whereas in cancer tissue the EGFr concentrations show an asymmetrical distribution (Figure 3).

On the basis of EGFr distribution in normal lung tissue samples we calculated a normal/pathologic cut-off point, which resulted in 12.9 fmoles/mg m.p. (mean of EGFr concentration in normal lung + 2 s.d.). EGFr in lung cancer was therefore evaluated both as a continuous quantitative parameter and as a dichotomic variable (positive/negative).

EGFr levels in 41/51 cancer samples were higher than in the normal tissue of the same patient (Table II). Fifty-seven per cent of cases showed EGFr levels above the cut-off point and could be therefore considered as EGFr positive.

**Figure 1** EGFr of typical Scatchard plot in lung cancer membrane. High affinity sites: 221 fmol ml$^{-1}$; Kd: 0.13 nM. Low affinity sites: 671 fmol ml$^{-1}$; Kd: 3.09 nM.

**Table I** EGFr concentrations in lung membranes (fmoles mg$^{-1}$ of membrane protein)

| | *Normal* | *Cancer* |
|---|---|---|
| Mean | 7.4 | 23.5 |
| s.d. | 2.7 | 27.0 |
| Median | 7.4 | 16.4 |
| Range | 1.9–13.4 | 1.5–176 |
| Number of cases | 51 | 51 |

Wilcoxon rank-sum test: $P = 0.0002$.

No significant relationships were found between EGFr and histologic type, lymph node status, clinical stage and pT (Table III). A trend for a direct relation between receptor positivity and grading were found (Table III).

**Figure 2** EGFr distribution in normal lung tissue. Kolmogorov-Smirnov test: $P = 0.988$.

**Figure 3** EGFr distribution in lung cancer tissue. Kolmogorov-Smirnov test: $P = 0.02$.

**Table II** EGFr in lung cancer tissue. Relationship to normal tissue

| | | *EGFr*[a] | | | | *EGFr*[a] | |
|---|---|---|---|---|---|---|---|
| *Cases* | *Histology* | *N* | *C* | *Cases* | *Histology* | *N* | *C* |
| P.L. | A | 3.6 | 8.1 | L.B. | A | 9.8 | 16.4 |
| R.P. | SCC | 5.7 | 23.8 | C.S. | A | 9.1 | 36.4 |
| P.B. | SCC | 8.0 | 12.7 | R.G. | SCC | 4.6 | 45.0 |
| M.G. | SCC | 2.2 | 4.6 | P.S. | SCC | 7.0 | 14.9 |
| C.G. | A | 9.8 | 22.4 | C.A. | U | 9.4 | 9.7 |
| S.S. | U | 7.2 | 3.1 | T.L. | A | 11.8 | 10.3 |
| V.G. | SCC | 8.1 | 45.4 | D.G. | SCC | 7.4 | 8.7 |
| S.M. | SCC | 8.3 | 24.9 | P.L. | A | 9.0 | 27.5 |
| D.R. | A | 4.1 | 19.7 | P.A. | A | 7.1 | 35.7 |
| F.O. | SCC | 8.7 | 16.9 | C.S. | LC | 5.9 | 40.0 |
| G.E. | SCC | 2.5 | 25.0 | M.O. | SCC | 6.4 | 15.5 |
| C.M. | U | 5.2 | 3.4 | S.I. | SCC | 6.5 | 8.8 |
| M.G. | LC | 10.1 | 18.1 | V.E. | SCC | 7.4 | 65.7 |
| O.A. | A | 10.8 | 6.0 | B.C. | SCC | 12.0 | 32.1 |
| P.W. | SCC | 7.2 | 24.1 | G.M. | LC | 8.2 | 13.6 |
| P.B. | SCC | 8.0 | 41.3 | F.C. | SCC | 13.4 | 3.2 |
| R.G. | A | 11.0 | 4.4 | O.G. | SCC | 11.4 | 26.5 |
| O.A. | SCC | 9.7 | 7.6 | D.M. | SCC | 4.7 | 8.0 |
| P.G. | SCC | 5.5 | 37.5 | F.G. | SCC | 12.1 | 75.1 |
| G.I. | SCC | 5.7 | 29.0 | F.B. | SCC | 7.9 | 6.4 |
| G.L. | A | 6.9 | 1.5 | B.G. | U | 8.9 | 36.5 |
| Z.A. | SCC | 4.9 | 5.2 | S.E. | LC | 4.9 | 12.1 |
| F.G. | A | 1.9 | 7.0 | S.A. | SCC | 9.0 | 175.6 |
| B.C. | SCC | 5.2 | 35.9 | F.G. | SCC | 7.1 | 9.9 |
| G.G. | U | 3.0 | 9.8 | B.M. | U | 7.5 | 9.1 |
| C.I. | A | 3.0 | 17.0 | | | | |

SCC: Squamous cell carcinomas; A: adenocarcinoma; LC: large cells carcinoma; U: unknown; N: normal tissue; C: cancer tissue.
[a]fmoles mg$^{-1}$ membrane protein.

**Table III** EGFr in lung cancer. Relationship to clinical and pathological parameters

| | Number of cases | | | EGFr Median fmoles mg$^{-1}$ m.p. | Interquartile range | P |
|---|---|---|---|---|---|---|
| | + | − | P | | | |
| Stage | | | | | | |
| 1 | 14 | 12 | | 14.5 | 8.5–35.6 | |
| 2 | 3 | 0 | 0.30 | 23.8 | — | 0.51 |
| 3 | 8 | 5 | | 16.9 | 6.6–30.3 | |
| Histology | | | | | | |
| Squamous | 18 | 10 | | 23.9 | 8.7–36.9 | |
| Adenocarcinomas | 7 | 6 | 0.46 | 16.4 | 6.5–24.9 | 0.35 |
| Large cell | 3 | 1 | | 15.8 | 12.5–34.5 | |
| Grading | | | | | | |
| G1 | 0 | 3 | | 9.7 | — | |
| G2 | 18 | 13 | 0.06 | 16.4 | 8.1–27.5 | 0.11 |
| G3 | 11 | 4 | | 25.0 | 12.1–37.5 | |
| Lymph node | | | | | | |
| 0 | 16 | 14 | | 14.5 | 8.1–32.9 | |
| ⩾1 | 11 | 4 | 0.20 | 17.0 | 9.7–35.7 | 0.50 |
| pT | | | | | | |
| 1 | 4 | 3 | | 16.4 | 6.4–27.5 | |
| 2 | 17 | 13 | 0.85 | 16.2 | 9.0–35.6 | 0.87 |
| 3 | 5 | 3 | | 21.0 | 3.5–37.0 | |

### Discussion

In the present investigation the evaluation of EGFr has been carried out in both lung cancer and histologically proven normal lung tissue samples, collected from lung bearing the tumour.

The number of normal lung tissue evaluated in the present study is higher than the total number of cases reported in so far published studies (Hwang *et al.*, 1986; Veale *et al.*, 1987; Veale *et al.*, 1989). It was therefore possible to compare both the binding characteristics and the distribution of EGFr concentrations between cancer and normal tissue using a number of cases adequate for statistical evaluation.

The binding characteristics of EGFr were similar in normal tissue and in cancer, showing a presence of two binding sites with different affinities, as previously noted both in cell cultures (Schlessinger, 1988; King & Cuatrecasas, 1982) and in lung tissues (Veale *et al.*, 1989).

From the binding studies we could determine the $^{125}$I-EGF concentration capable of saturating the high affinity sites (0.5 nM), which can be used in a single saturating dose assay when small tissue samples are available.

In the cancer tissue receptor levels (range 1.5–176 fmol mg$^{-1}$ of protein) are higher than in normal lung (range 1.9–13.4 fmol mg$^{-1}$ of protein). These findings confirm preliminary observations reported by Hwang *et al.* (1986) on six cases, by Veale *et al.* (1987) on 17 cases and by Veale *et al.* (1989) on eight cases. In 80% of cases the concentrations in cancer are higher than in the normal tissue from the same patient. Considering that EGFr concentrations were found to be very high in fetal lung (Nexo & Kryger-Baggesen, 1989), the increased expression of EGFr in lung cancer could be a consequence of tissue dedifferentiation, and could regulate the tumour growth by an autocrine mechanism.

The distribution pattern in normal lung tissue was Gaussian, suggesting that EGFr in lung tissue could be physiologically expressed. This finding allows for the calculation of a normal/pathologic cut-off point that could be used to classify cancer samples even when the normal tissue is not available.

We did not find significant differences in EGFr concentrations between squamous cell carcinomas, adenocarcinomas and large cell carcinomas, which is in agreement with the previous studies carried out with binding methods (Hwang *et al.*, 1986; Veale *et al.*, 1989). Instead, immunohistochemical studies report conflicting results. Hendler *et al.* (1984) found EGFr only in squamous cell carcinomas. Other authors found EGFr in all the NSCLCs, with higher concentrations in squamous carcinomas (Berger *et al.*, 1987; Sobol *et al.*, 1987; Veale *et al.*, 1987) or without significative differences between histological types (Cerny *et al.*, 1986; Dazzi *et al.*, 1989). However, immunohistochemical methods do not allow for a precise quantification and, in particular for EGFr, seems to be less sensitive to binding saturation assays (Sobol *et al.*, 1987; Veale *et al.*, 1989).

The attempt to establish the relationships between EGFr and other known prognostic factors led to conflicting results. Higher concentrations in stage III tumours have been found (Veale *et al.*, 1987), but these results were not confirmed by the same authors in a following report carried out by a saturation binding assay (Veale *et al.*, 1989). Dazzi *et al.* (1989), in a retrospective study performed by immunohistochemical method, found a higher expression of EGFr in well differentiated tumour.

However, the EGFr positivity was so far established on the basis of the sensitivity of assays or semiquantitative classifications.

In this study, the finding of Gaussian distribution in normal tissue allowed us to define the EGFr positivity in lung cancer on the basis of a physiologically and statistically acceptable cut-off point. No relationships were found between EGFr concentrations or positivity rates and the prognostic parameters evaluated. A possible independent prognostic role of EGFr could be therefore postulated.

The present investigation was financially supported in part by the Regione Veneto, Italy and by the Italian Association for Cancer Research (A.I.R.C.), Milan, Italy.

### References

BERGER, M.S., GULLICK, W.J., GREENFIELD, C., EVANS, S., ADDIS, B.J. & WATERFIELD, M.D. (1987). Epidermal growth factor receptors in lung tumours. *J. Pathol.*, **152**, 297.

BRADFORD, M. (1976). A rapid and sensitive method for the quantification of microgram quantities of protein utilizing the principle of protein dye-binding. *Anal. Biochem.*, **72**, 248.

CARPENTER, G. (1983). The biochemistry and physiology of the receptor-kinasi for epidermal growth factor. *Mol. Cell. Endocrinol.*, **31**, 1.

CARPENTER, G. & COHEN, S. (1979). Epidermal growth factor. *Ann. Rev. Biochem.*, **48**, 193.

CERNY, T., BARNES, D.M., HASLETON, P. & 4 others (1986). Expression of epidermal growth factor receptor (EGF-R) in human lung tumours. *Br. J. Cancer*, **54**, 265.

COOPER, J.A., BOWEN-POPE, D.F., RAINES, E., ROSS, R. & HUNTER, T. (1982). Similar effects of platelet-derived growth factor and epidermal growth factor on the phosphorylation of tyrosine in cellular proteins. *Cell*, **31**, 263.

DAZZI, H., HALESTON, P.S., THATCHER, N. & 4 others (1989). Expression of epidermal growth factor receptor (EGF-R) in non-small cell lung cancer. Use of archival tissue and correlation of EGF-R with histology, tumour size, node status and survival. *Br. J. Cancer*, **59**, 746.

DITTADI, R., GION, M., BRAZZALE, A. & BRUSCAGNIN, G. (1990). Radioligand binding assay of epidermal growth factor receptor: causes of variability and standardization of the assay. *Clin. Chem.*, **36**, 849.

DOWNWARD, J., PARKER, P. & WATERFIELD, M.D. (1984). Autophosphorylation sites on the epidermal growth factor receptor. *Nature*, **311**, 483.

FELDMAN, H.A. (1972). Mathematical theory of complex ligand-binding systems of equilibrium: some methods for parameter fitting. *Anal. Biochem.*, **48**, 317.

GAMOU, S., HUNTS, J., HARIGAI, H. & 4 others (1987). Molecular evidence for the lack of epidermal growth factor receptor gene expression in small cell lung carcinoma cells. *Cancer Res.*, **47**, 2668.

GILL, G.N., BERTICS, P.J. & SANTON, J.B. (1987). Epidermal growth factor and its receptor. *Mol. Cell. Endocrinol.*, **51**, 169.

HENDLER, F.J. & OZANNE, B.W. (1984). Human squamous cell lung cancers express increased epidermal growth factor receptors. *J. Clin. Invest.*, **74**, 647.

HUNTER, T. & COOPER, J.A. (1981). Epidermal growth factor induces rapid tyrosine phosphorylation of proteins in A431 human tumor cells. *Cell*, **24**, 741.

HWANG, D.L., TAY, Y.-C., LIN, S.S. & LEV-RAN, A. (1986). Expression of epidermal growth factor receptors in human lung tumors. *Cancer*, **58**, 2260.

ISHITOYA, J., TORIYAMA, M., OGUCHI, N. & 4 others (1989). Gene amplification and overexpression of EGF receptor in squamous cell carcinomas of the head and neck. *Br. J. Cancer*, **59**, 559.

KING, A.C. & CUATRECASAS, P. (1982). Resolution of high and low affinity epidermal growth factor receptors. *J. Biol. Chem.*, **257**, 3053.

LIBERMANN, T.A., RAZON, N., BARTAL, A.D., YARDEN, Y., SCHLESSINGER, J. & SOREQ, H. (1984). Expression of epidermal growth factor receptors in human brain tumors. *Cancer Res.*, **44**, 753.

NEAL, D.E., MARSH, C., BENNETT, M.K. & 4 others (1985). Epidermal growth factor receptors in human bladder cancer: comparison of invasive and superficial tumors. *Lancet*, **i**, 366.

NEXO, E. & KRYGER BAGGESEN, N. (1989). The receptor for epidermal growth factor is present in fetal kidney, liver and lung. *Regul. Pept.*, **26**, 1.

NICKELL, K.A., HALPER, J. & MOSES, H.L. (1983). Transforming growth factors in solid human malignant neoplasms. *Cancer Res.*, **43**, 1966.

REYNOLDS, F.H.J., TODARO, G.J., FRYLING, C. & STEPHENSON, J.R. (1981). Human transforming growth factor induce tyrosine phosphorylation of EGF receptors. *Nature*, **292**, 259.

SAINSBURY, J.R.C., FARNDON, J.R., NEEDHAM, G.K., MALCOLM, A.J. & HARRIS, A.L. (1987). Epidermal growth factor receptor status as predictor of early recurrence of and death from breast cancer. *Lancet*, **i**, 1398.

SCHLESSINGER, J. (1988). The epidermal growth factor receptor as a multifunctional allosteric protein. *Am. Chem. Soc.*, **27**, 3119.

SOBOL, R.E., ASTARITA, R.W., HOFEDITZ, C. & 4 others (1987). Epidermal growth factor receptor expression in human lung carcinomas defined by monoclonal antibody. *J. Natl Cancer Inst.*, **79**, 403.

UICC (1979). *TNM Classification of Malignant Tumours*. Ed. M.H. Harmer: Geneva.

VEALE, D., ASHCROFT, T., MARSH, C., GIBSON, G.J. & HARRIS, A.L. (1987). Epidermal growth factor receptors in non-small cell lung cancer. *Br. J. Cancer*, **55**, 513.

VEALE, D., KERR, N., GIBSON, G.J. & HARRIS, A.L. (1989). Characterisation of epidermal growth factor receptor in primary human non-small cell lung cancer. *Cancer Res.*, **49**, 1313.

WHO (1981). *Histological Typing of Lung Tumours*, 2nd edn. WHO: Geneva.